# Observed readmission timing and diagnostic profiles among high-risk adolescents and general child and adolescent inpatients: a 10-year cohort study

**DOI:** 10.3389/fpsyt.2026.1862976

**Published:** 2026-10-16

**Authors:** Gyula Sófi

**Affiliations:** Kertváros Pszichológiai Rendelő, Budapest, Hungary

**Keywords:** adolescent psychiatry, clinical instability, developmental psychopathology, externalizing psychopathology, inpatient care, post-discharge risk, psychiatric rehospitalization, time-to-readmission

## Abstract

**Background:**

Rehospitalization is a key indicator of clinical instability in child and adolescent psychiatric populations, yet most studies focus on its occurrence rather than its timing and diagnostic context. It remains unclear whether high-risk adolescents and general child and adolescent inpatient populations show distinct patterns of clinical instability beyond differences in rehospitalization frequency.

**Methods:**

We conducted a retrospective cohort study using a 10-year inpatient dataset from a national child and adolescent psychiatric center. A general inpatient cohort (N = 4016) was compared with a high-risk cohort (N = 570) defined by at least one police- or justice-related admission during the observation period. Rehospitalization rates, observed time to first readmission, length of stay, and diagnostic categories were examined.

**Results:**

Admission-frequency distributions were broadly comparable between cohorts and did not differ significantly overall. Among rehospitalized patients, the median observed time to first readmission was 19 days in the high-risk cohort and 190 days in the general inpatient cohort. Length of stay was comparable between cohorts. Diagnostic-category distributions differed significantly between cohorts (χ²(3) = 2038.04, p < 0.001; Cramér’s V = 0.667), with externalizing and substance-related disorders predominating in the high-risk cohort (76.7%) and developmental–emotional disorders predominating in the general inpatient cohort (66.7%).

**Conclusions:**

The two clinically defined inpatient cohorts showed different patterns of rehospitalization and diagnostic characteristics. Among rehospitalized patients, the observed median interval to first readmission was shorter in the high-risk cohort; however, this descriptive difference should be interpreted in the context of substantial baseline age differences and unequal opportunities for observing readmission within pediatric psychiatric care. These findings highlight the potential clinical relevance of considering observed readmission timing alongside diagnostic characteristics, while recognizing the limitations of unadjusted retrospective comparisons.

## Introduction

Child and adolescent psychiatric inpatient populations are characterized by marked clinical heterogeneity, encompassing a broad spectrum of developmental, emotional, and behavioral disorders. Within these populations, rehospitalization is commonly used as an indicator of clinical instability, treatment outcomes, and service utilization. Previous studies have identified multiple factors associated with readmission in youth, including symptom severity, comorbidity, trauma exposure, and family-related variables (1–5). However, these studies have focused predominantly on identifying predictors of whether readmission occurs, rather than examining when it occurs.

Despite this growing body of research, comparatively little attention has been paid to the timing of psychiatric readmission. This represents an important gap in the literature, as increasing evidence suggests that the period immediately following psychiatric discharge is associated with substantially elevated risk for adverse outcomes, including suicide and premature mortality (6–11). Furthermore, early post-discharge follow-up has been shown to mitigate this risk, highlighting the clinical importance of the early post-discharge period. (12). Beyond estimating readmission rates, understanding when rehospitalization occurs and which diagnostic profiles characterize different inpatient populations has direct clinical relevance. Characterizing readmission timing alongside diagnostic profiles may provide additional information relevant to post-discharge assessment, the timing of follow-up care, and the development of more targeted interventions for vulnerable adolescents.

At the same time, child and adolescent inpatient populations are not diagnostically homogeneous. General psychiatric inpatient cohorts are typically characterized by a predominance of developmental and emotional disorders, whereas subgroups of high-risk youth – particularly those with offending behavior or involvement with the justice system – show markedly elevated rates of externalizing psychopathology, substance use disorders, and complex comorbidity patterns. Meta-analytic evidence has demonstrated a high prevalence of conduct disorder, substance use, and trauma-related conditions in justice-involved adolescents, reflecting a highly burdened clinical profile (13).

These observations raise an important conceptual question: Do high-risk and general psychiatric inpatient populations differ only in the frequency of rehospitalization, or are differences also observable in readmission timing and diagnostic characteristics?

From a developmental psychopathology perspective, high-risk behaviors in adolescence – particularly those associated with externalizing disorders and substance use – are understood as the cumulative outcome of neurodevelopmental vulnerability, trauma exposure, family dysfunction, and social marginalization. Accordingly, the contrasting diagnostic characteristics of these populations may be associated with different patterns of post-discharge clinical instability.

In this context, there is a need for studies that move beyond aggregate rehospitalization rates and explicitly examine rehospitalization frequency, observed time-to-readmission, and diagnostic profiles across clinically defined child and adolescent subgroups. The present study addresses this gap by comparing a high-risk adolescent cohort with a general child and adolescent inpatient population across these three dimensions. Specifically, we compared rehospitalization rates, observed time to first readmission, and diagnostic profiles across the two cohorts. We hypothesized that, although overall rehospitalization rates would differ only modestly, the two cohorts would show different descriptive patterns of observed time to first readmission and diagnostic characteristics.

In addition to addressing these aims, the present study has broader international relevance, as it examines structural features of psychiatric instability that are not specific to a single healthcare system but reflect generalizable developmental and clinical processes. By descriptively examining observed readmission intervals alongside diagnostic characteristics, the present study may provide clinically relevant information beyond rehospitalization rates alone and generate hypotheses for future studies using prospectively defined follow-up and adjusted time-to-event analyses.

## Methods

### Study design and setting

This retrospective cohort study was conducted at the Department of Child and Adolescent Psychiatry of the Heim Pál National Institute of Pediatrics (Budapest, Hungary). Clinical data were retrospectively extracted from the institution’s electronic medical records covering a 10-year study period. The study was approved by the Institutional Research Ethics Committee (KUT-27/2021, IKEB).

### Study population

Two cohorts were analyzed. The general inpatient cohort comprised children and adolescents who did not meet the predefined criteria for police- or justice-related involvement during the study period (N = 4016). The high-risk cohort (N = 570) consisted of adolescents admitted under police- or justice-related circumstances. These admissions included direct police escorts to psychiatric care, referrals originating from juvenile justice procedures, court-ordered psychiatric inpatient assessments, and other admissions with documented police or judicial involvement in the clinical record. Thus, the high-risk cohort was defined by the predefined inclusion criteria and was not restricted to, or defined solely by, direct police escort. The cohort was previously described in a separate publication (14); however, the present study uses these data within a new comparative framework focusing on readmission timing and diagnostic profiles.

Both cohorts originated from the same institution, covered the same observation period, and were derived from the same electronic medical record system. Comparative analyses were restricted to variables available in both datasets. Any structural differences between datasets are explicitly described where relevant and considered in the interpretation of findings.

Patients were eligible if they had at least one psychiatric inpatient admission during the study period. Cases involving non-patient admissions (e.g., accompanying caregivers) were excluded based on clinical and administrative indicators (age, diagnostic coding, and discharge notes).

Cohort membership was assigned retrospectively at the individual-patient level across the complete 10-year observation period. An anonymized patient record was classified as high-risk if at least one hospitalization during the study period met the predefined police- or justice-related criteria. Once this criterion was met, all recorded psychiatric hospitalizations linked to that anonymized patient during the observation period were retained as part of that patient’s hospitalization history within the high-risk cohort, including admissions occurring before the police- or justice-related event and admissions without documented police or justice involvement. Patients for whom no such circumstance was identified during the observation period constituted the general inpatient cohort.

Accordingly, high-risk status represented a retrospective patient-level classification and did not define the temporal origin of the readmission analysis. For both cohorts, demographic and diagnostic variables used in the comparative analyses were derived from the first observed hospitalization during the study period. Admission-frequency categories reflected the cumulative number of inpatient episodes recorded for each patient over the observation period.

Following discharge, patients generally continued their care through outpatient child and adolescent psychiatric services. Medication adjustments were followed by scheduled outpatient reviews according to clinical need. Patients requiring more intensive rehabilitation could be referred to the hospital’s day-care psychiatric program, and child protection services were involved whenever clinically or socially indicated. The timing and intensity of follow-up were determined by the treating clinical team according to individual clinical needs.

### Relationship to prior publication

This study builds upon our previously published analysis of high-risk adolescents admitted under police- or justice-related circumstances (14), which provided a detailed descriptive characterization of psychiatric morbidity, comorbidity, and readmission patterns within this subgroup.

The present study addresses a distinct research question by introducing a comparative framework that incorporates a general inpatient cohort and focuses specifically on readmission timing and diagnostic profiles across clinically defined populations. While the high-risk cohort partially overlaps with the previously published dataset, the current analyses, outcomes, and interpretation are conceptually and methodologically different, emphasizing between-group comparison and observed rehospitalization characteristics rather than cohort-specific description.

The present comparative analyses and between-cohort statistical tests were not reported in the prior publication.

### Measures

#### Rehospitalization and admissions

Rehospitalization was defined as the occurrence of two or more inpatient admissions during the observation period, consistent with prior adolescent psychiatric research (3). The total number of admissions per patient was calculated, and patients were classified into three admission-frequency categories: one admission, two admissions, and three or more admissions.

#### Time-to-readmission

Among patients with at least one observed rehospitalization, time to first readmission was defined as the interval, in days, between discharge from the first observed hospitalization during the study period and admission for the second observed hospitalization. For patients with multiple admissions, only this first readmission interval was analyzed; later inter-admission intervals were not included. Median observed time to first readmission was calculated separately for each cohort. High-risk cohort membership was assigned retrospectively at the patient level and did not determine the temporal origin of this interval.

Because follow-up was limited to readmissions recorded within the participating child and adolescent psychiatric institution, the opportunity to observe subsequent readmission varied according to age at the first observed hospitalization and ended with transition out of pediatric care. Furthermore, the analysis was conditional on an observed rehospitalization. The reported intervals were therefore treated as descriptive characteristics of recorded readmissions rather than as adjusted estimates of between-cohort differences in time to rehospitalization, and no formal between-group hypothesis test was performed for this variable.

#### Length of stay

Length of stay was defined as the number of inpatient days during the first observed hospitalization for each patient. To ensure one independent observation per individual, LOS comparisons between cohorts were based on this first observed hospitalization. Cohort-specific median and mean values were calculated, and LOS distributions were compared using a two-sided Mann–Whitney U test.

#### Diagnostic classification

For both cohorts, diagnostic comparisons were based on the principal discharge diagnosis recorded at the first observed hospitalization during the study period. Diagnoses were coded according to ICD-10 and assigned to one of four study-defined diagnostic categories: (1) externalizing and substance-related disorders, (2) internalizing disorders, (3) developmental–emotional disorders, and (4) other psychiatric disorders. Each patient was assigned to a single category according to the recorded principal discharge diagnosis, resulting in mutually exclusive diagnostic classification in both cohorts. No *post hoc* clinical hierarchy was imposed among co-occurring diagnoses.

The four-category grouping was developed specifically for the present comparative analysis to permit comparison of broad diagnostic patterns across the two cohorts and should not be interpreted as a validated psychiatric taxonomy. The ICD-10 mapping used to assign principal discharge diagnoses to the four study-defined categories is provided in **Supplementary Table S1**.

#### Psychometric assessment

As part of routine inpatient clinical assessment, standardized psychometric instruments, including the Mini International Neuropsychiatric Interview for Children and Adolescents (MINI-KID) (15) and the Strengths and Difficulties Questionnaire (SDQ) (16), were administered to support diagnostic evaluation. These instruments informed routine clinical decision-making but were not included in the statistical analyses of the present study.

### Statistical analysis

Descriptive statistics were used to summarize demographic and clinical characteristics. Continuous variables are presented as mean ± standard deviation (SD) or median values, as appropriate. Categorical variables are presented as counts and percentages.

Between-group comparisons of sex and admission-frequency categories were performed using Pearson’s chi-square test. Age was compared using Welch’s t-test because of unequal variances between cohorts, and LOS was compared using a two-sided Mann–Whitney U test. Rank-biserial correlation was calculated as an effect-size measure for the LOS comparison. Diagnostic-category distributions were compared between cohorts using Pearson’s chi-square test, and Cramér’s V was calculated as an effect-size measure. Time to first readmission was similarly summarized descriptively using median observed intervals among patients who experienced at least one rehospitalization, without formal between-group hypothesis testing.

Analyses were conducted using IBM SPSS Statistics for Windows, Version 26.0 (IBM Corp., Armonk, NY, USA).

The objective of the present study was not to compare demographically matched cohorts or to estimate the independent effect of justice involvement. Rather, the analyses were designed to descriptively characterize two clinically defined inpatient populations as encountered in routine psychiatric practice, including their diagnostic profiles and the observed readmission intervals captured within the available dataset.

## Results

### Sample characteristics

The general inpatient cohort comprised 4016 children and adolescents, whereas the high-risk cohort comprised 570 adolescents. The high-risk cohort contained a significantly greater proportion of males (72.1% vs. 56.3%, p < 0.001) and was significantly older (15.3 ± 2.1 vs. 11.8 ± 3.3 years, p < 0.001) than the general inpatient cohort.

### Rehospitalization and admissions

Most patients in both cohorts experienced only one psychiatric inpatient admission during the observation period (79.0% vs. 75.0%). The distribution of admission frequencies was broadly similar across cohorts, with 13.5% versus 15.2% of patients experiencing exactly two admissions and 7.5% versus 9.6% experiencing three or more admissions in the general inpatient and high-risk cohorts, respectively. Overall differences in admission-frequency distribution did not reach statistical significance (χ²(2)=5.10, p=0.078).

### Observed time to first readmission

Among patients who experienced at least one rehospitalization, the median observed time to first readmission was 190 days in the general inpatient cohort and 19 days in the high-risk cohort (**Table 1**). These findings were not subjected to formal between-group hypothesis testing.

**Table 1:** Demographic and clinical characteristics of the general child and adolescent psychiatric inpatient and high-risk adolescent cohorts.

Variable	General inpatient (N = 4016)	High-risk cohort (N = 570)	p-value
Sex, n (%)			<0.001
Male	2262 (56.3%)	411 (72.1%)	
Female	1754 (43.7%)	159 (27.9%)	
Age, years (mean ± SD)	11.8 ± 3.3	15.3 ± 2.1	<0.001
Number of admissions			0.078
Single admission, n (%)	3173 (79.0%)	428 (75.0%)	
2 admissions, n (%)	542 (13.5%)	87 (15.2%)	
≥3 admissions, n (%)	301 (7.5%)	55 (9.6%)	
Observed time to first readmission, days			–
Median	190	19	
Length of stay (LOS), days			0.645
Median	8.0	7.0	–
Mean	8.5	8.5	–
Diagnostic categories, %			<0.001
Externalizing/substance-related	6.0%	76.7%	
Internalizing	9.0%	11.2%	
Developmental–emotional	66.7%	9.3%	
Other	18.3%	2.8%	

χ² comparison across admission-frequency categories: p = 0.078. Diagnostic-category distributions were compared using Pearson’s chi-square test (χ²(3) = 2038.04, p < 0.001; Cramér’s V = 0.667).

Observed time to first readmission was calculated among patients who experienced at least one rehospitalization and was defined as the interval between discharge from the first observed hospitalization and admission for the second observed hospitalization during the study period.

‘-’ indicates not applicable.

Values are presented as n (%) or mean ± SD unless otherwise indicated.

P-values are presented for variables for which formal between-group comparisons were considered appropriate. Chi-square tests were used for sex, admission-frequency categories, and diagnostic-category distributions; Welch’s t-test was used for age. LOS was compared using a two-sided Mann–Whitney U test (U = 1,158,154.5, p = 0.645; rank-biserial r = 0.012). Cramér’s V was calculated as an effect-size measure for the diagnostic-category comparison.

Both cohorts represent unique individuals rather than admissions. Admission-frequency categories indicate the cumulative number of psychiatric inpatient admissions recorded per individual during the observation period.

Among rehospitalized patients, the median observed time to first readmission was 190 days in the general inpatient cohort and 19 days in the high-risk cohort. Diagnostic-category distributions differed significantly between cohorts (χ²(3) = 2038.04, p < 0.001; Cramér’s V = 0.667), with externalizing and substance-related disorders predominating in the high-risk cohort and developmental–emotional disorders predominating in the general inpatient cohort.

### Length of stay

Length of stay did not differ significantly between cohorts. The median LOS was 8.0 days in the general inpatient cohort and 7.0 days in the high-risk cohort, whereas the mean LOS was 8.5 days in both cohorts (Mann–Whitney U = 1,158,154.5, p = 0.645; rank-biserial r = 0.012).

### Diagnostic categories

Diagnostic-category distributions differed significantly between the two cohorts (χ²(3) = 2038.04, p < 0.001; Cramér’s V = 0.667; **Figure 1**). In the general inpatient cohort, developmental–emotional disorders were the most frequent principal discharge diagnostic category (66.7%), followed by other psychiatric disorders (18.3%), internalizing disorders (9.0%), and externalizing and substance-related disorders (6.0%). In the high-risk cohort, externalizing and substance-related disorders were the predominant principal discharge diagnostic category (76.7%), followed by internalizing disorders (11.2%), developmental–emotional disorders (9.3%), and other psychiatric disorders (2.8%).

**Figure 1:**
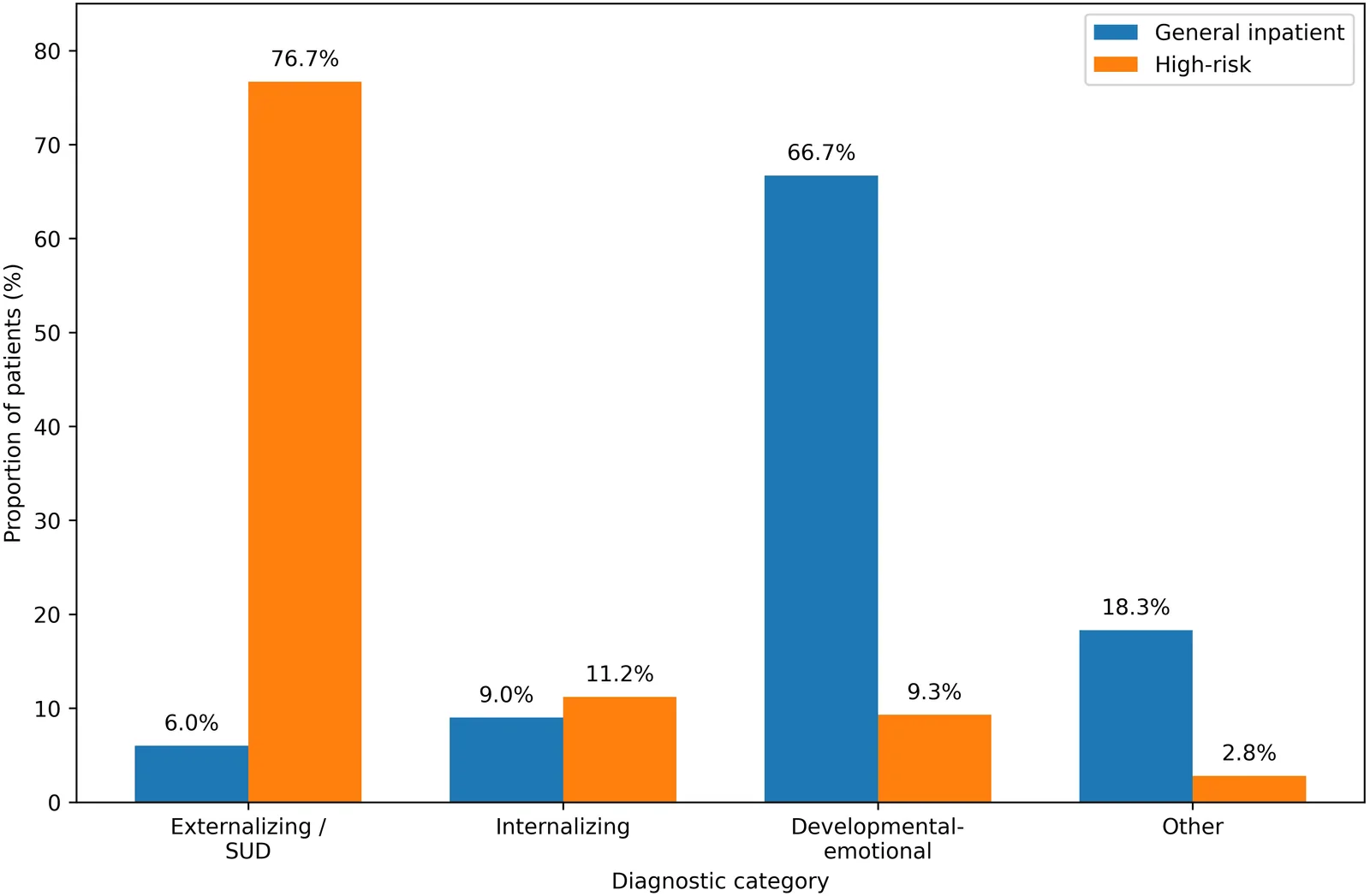
Distribution of diagnostic categories in the general child and adolescent psychiatric inpatient and high-risk adolescent cohorts. For both cohorts, categories were based on the principal discharge diagnosis recorded at the first observed hospitalization during the study period. Each patient was assigned to one mutually exclusive study-defined diagnostic category according to the ICD-10 mapping presented in **Supplementary Table S1**. Sample sizes: N = 4016 (general inpatient cohort) and N = 570 (high-risk cohort). Diagnostic-category distributions differed significantly between cohorts (χ²(3) = 2038.04, p < 0.001; Cramér’s V = 0.667).

## Discussion

### Principal findings

The present study extends previous work on high-risk adolescent psychiatric populations by descriptively examining rehospitalization frequency, observed readmission intervals, and diagnostic characteristics in two clinically defined inpatient cohorts. Admission-frequency distributions were broadly comparable, whereas the observed median interval to first readmission among rehospitalized patients was 19 days in the high-risk cohort and 190 days in the general inpatient cohort. Because the cohorts differed substantially in baseline age and therefore in the opportunity for subsequent readmission to be observed within pediatric care, these intervals should be interpreted as descriptive characteristics of the available dataset rather than as adjusted evidence of an independent between-cohort difference in readmission timing.

Rehospitalization rates alone may not fully capture clinically meaningful differences between child and adolescent psychiatric inpatient populations, despite being the primary outcome reported in most previous studies (3, 5, 17). Although the observed intervals cannot establish an independent cohort effect, documenting when recorded readmissions occur may provide clinically relevant descriptive information alongside rehospitalization frequency. This finding is consistent with evidence demonstrating that the immediate post-discharge period is associated with substantially elevated risk for adverse outcomes, including suicide and premature mortality (8–10).

### Observed readmission intervals in clinical context

The observed readmission intervals occurred in the context of markedly different clinical characteristics. The clinical characteristics of the high-risk cohort are consistent with previous research on justice-involved adolescents, in whom externalizing psychopathology, substance use, and psychiatric comorbidity are highly prevalent (13, 18). Developmental research has similarly identified externalizing pathways associated with adolescent substance use and behavioral risk (19). Furthermore, studies of justice-involved populations indicate increased psychiatric morbidity and service utilization following transitions out of structured or supervised settings (20). These findings provide relevant clinical context for the high-risk population examined in the present study, but they should not be interpreted as explaining the observed 19-day median readmission interval in this cohort.

However, the present descriptive data do not establish whether diagnostic characteristics, justice-related circumstances, age, service factors, or differences in the available observation window account for the observed readmission intervals.

More broadly, the contrasting clinical characteristics observed across the two cohorts illustrate the heterogeneity of child and adolescent psychiatric inpatient populations. However, because the present descriptive data cannot establish an independent association between cohort characteristics and readmission timing, risk stratification should not be inferred from the observed 19-day versus 190-day difference alone. Rather, the findings should be interpreted alongside the broader evidence supporting individualized post-discharge assessment and follow-up strategies for vulnerable psychiatric populations (12).

### Readmission timing and the early post-discharge period

Among rehospitalized patients, the observed median interval to first readmission was 19 days in the high-risk cohort and 190 days in the general inpatient cohort. This large descriptive difference is clinically notable but cannot be interpreted as evidence that membership in the high-risk cohort independently predicts earlier readmission. In particular, the older age of the high-risk cohort reduced the period during which subsequent admissions could be observed within pediatric psychiatric care, and admissions after transition to adult services were unavailable in the present dataset.

More broadly, previous research indicates that the period immediately following psychiatric discharge represents a phase of heightened vulnerability, characterized by elevated risks of suicide, relapse, and service re-entry (8–10).

The present data do not identify the mechanisms underlying the observed readmission intervals. Previous studies of justice-involved youth have identified persistent environmental instability, limited continuity of care, behavioral dysregulation, and substance use as factors potentially contributing to psychiatric morbidity and service utilization. This interpretation is supported by studies showing that justice-involved youth experience increased psychiatric morbidity and service utilization following transitions out of structured or supervised settings (20), as well as reduced engagement with outpatient treatment in the presence of co-occurring externalizing and substance use disorders (18).

The longer observed median interval in the general inpatient cohort should likewise be interpreted descriptively and not as evidence of an intrinsically different temporal course.

Regardless of the observed between-cohort difference, timely post-discharge follow-up remains clinically important for vulnerable adolescents, consistent with evidence that early post-discharge contact is associated with improved outcomes and reduced suicide risk (12).

### Diagnostic profiles and developmental pathways

Diagnostic-category distributions differed significantly between the two cohorts, with a large effect size. Externalizing and substance-related disorders constituted the principal discharge diagnostic category for 76.7% of the high-risk cohort at the first observed hospitalization, consistent with the high burden of externalizing psychopathology and substance use previously reported in justice-involved youth (13, 14). These conditions are frequently associated with impulsivity, behavioral dysregulation, and reduced treatment engagement, but the present study cannot determine their contribution to the observed readmission intervals. This interpretation is consistent with evidence that justice-involved adolescents frequently present with co-occurring psychiatric and substance use disorders, which complicate treatment engagement and continuity of care (18).

The contrasting diagnostic-category distributions provide clinical context for the two cohorts but do not establish a causal or independent relationship between diagnostic category and readmission timing.

At the cohort level, the predominance of externalizing and substance-related disorders in the high-risk cohort and developmental–emotional disorders in the general inpatient cohort occurred alongside different observed median readmission intervals; however, no causal or independent association between diagnostic characteristics and readmission timing can be inferred from these descriptive data.

### Clinical and service implications

The findings have potential relevance for clinical practice and service organization but should not be used to infer different follow-up requirements solely from the observed between-cohort readmission intervals. Nevertheless, the high clinical burden of the high-risk cohort and the broader evidence supporting timely post-discharge care suggest that structured follow-up, rapid access to outpatient services, and interventions addressing substance use, behavioral dysregulation, and environmental adversity remain clinically relevant considerations. Previous studies indicate that timely outpatient follow-up after psychiatric discharge is associated with improved outcomes, including reduced suicide risk (12), supporting the broader clinical importance of continuity of care after discharge.

### Strengths and limitations

A particularly important limitation concerns the unequal opportunity to observe readmission within pediatric psychiatric care. The high-risk cohort was substantially older at baseline than the general inpatient cohort (mean age 15.3 vs. 11.8 years) and therefore had, on average, a shorter period during which subsequent admissions could be captured before transition out of child and adolescent psychiatric services. Because the available retrospective dataset did not capture admissions to adult psychiatric services or other institutions, the observed 19-day and 190-day median intervals cannot be interpreted as adjusted estimates of an independent between-cohort difference in time to readmission. The magnitude of the descriptive difference may be influenced by this age-dependent observation-window constraint. Accordingly, the reported intervals are interpreted only as observed characteristics of rehospitalized patients within the available institutional dataset.

Several limitations should be considered when interpreting the findings of this study. First, the retrospective design precludes causal inference and relies on routinely collected clinical data, which may be subject to variability in diagnostic practices and documentation. In particular, reliance on a single principal discharge diagnosis likely underestimates the extent of psychiatric comorbidity, especially in high-risk populations where complex, overlapping symptom profiles are common.

Second, although diagnostic classification was harmonized by using the principal discharge diagnosis from the first observed hospitalization and the same mutually exclusive study-defined categories in both cohorts, the resulting comparison remains unadjusted. The cohorts differed substantially in age and sex distributions, and these differences may partly contribute to the observed diagnostic-category distributions. In addition, classification based on a single principal discharge diagnosis does not capture the full extent of psychiatric comorbidity, particularly in high-risk populations with complex co-occurring disorders (18).

Third, the study was conducted within a single national inpatient center, which may limit generalizability to other healthcare systems with different service structures, discharge practices, or community care availability. Accordingly, differences in admission thresholds, inpatient service organization, discharge practices, and the availability of community-based follow-up across healthcare systems may limit the direct transferability of the present findings to international settings. At the same time, the large, longitudinal dataset from a centralized tertiary child and adolescent psychiatric center enhances internal consistency and allows detailed characterization of clinically severe adolescent populations.

In addition, time to first readmission was analyzed descriptively rather than using formal survival analysis. Consequently, the reported median intervals should be interpreted as observed values among rehospitalized patients and not as population-based time-to-event estimates.

The retrospective data also did not allow reliable differentiation between clinically driven readmissions and admissions related to administrative, legal, or placement circumstances. This limitation is particularly relevant to the high-risk cohort and should be considered when interpreting its short median observed time to first readmission.

Furthermore, the high-risk definition encompassed heterogeneous police- and justice-related circumstances, including crisis-related police involvement, juvenile justice referrals, and court-ordered assessments; these subgroups may differ in clinical characteristics, service trajectories, and post-discharge environments.

Finally, follow-up was restricted to inpatient admissions recorded within the participating institution. Consequently, psychiatric readmissions occurring at other hospitals could not be identified. In addition, readmissions occurring after transition to adult psychiatric services were not captured, potentially leading to an underestimation of rehospitalization during longer-term follow-up.

## Conclusion

This retrospective study identified differences in rehospitalization characteristics and diagnostic-category distributions between two clinically defined child and adolescent psychiatric inpatient populations. Among rehospitalized patients, the median observed interval to first readmission was 19 days in the high-risk cohort and 190 days in the general inpatient cohort. These values describe readmissions captured within the available institutional dataset and should not be interpreted as adjusted evidence of an independent difference in readmission timing, particularly given substantial baseline age differences and unequal opportunities for follow-up within pediatric care.

Despite these limitations, the findings illustrate the potential value of examining rehospitalization beyond frequency alone and provide clinical observations that may inform future studies using prospectively defined follow-up periods, linked service data, and adjusted time-to-event methods.

## Data Availability

Anonymized data supporting the conclusions of this article are available from the corresponding author upon reasonable request, subject to applicable clinical data protection regulations and ethical restrictions.
