# Supplementary material for: Observed readmission timing and diagnostic profiles among high-risk adolescents and general child and adolescent inpatients: a 10-year cohort study
Source: Front Psychiatry. 2026 Oct 16;17:1862976. doi: 10.3389/fpsyt.2026.1862976 (PMC13720245; doi:10.3389/fpsyt.2026.1862976)
Supplement: Supplementary file 1 [file Table1.docx]

**Supplementary Table S1**. ICD-10 mapping of principal discharge diagnoses to the four study-defined diagnostic categories

| **Study diagnostic category** | **Included ICD-10 diagnoses** |
| --- | --- |
| **Externalizing and substance-related disorders** | Mental and behavioural disorders due to psychoactive substance use (F10–F19); hyperkinetic disorders (F90.xx); conduct disorders (F91.xx); mixed disorders of conduct and emotions (F92.xx) |
| **Internalizing disorders** | Mood [affective] disorders (F30–F39); neurotic, stress-related and somatoform disorders (F40–F48); other behavioural and emotional disorders with onset usually occurring in childhood and adolescence (F98.xx) |
| **Developmental–emotional disorders** | Behavioural syndromes associated with physiological disturbances and physical factors (F50–F59); intellectual disabilities (F70–F79); disorders of psychological development (F80–F89); emotional disorders with onset specific to childhood (F93.xx); disorders of social functioning with onset specific to childhood and adolescence (F94.xx); tic disorders (F95.xx) |
| **Other psychiatric disorders** | Other mental disorders due to known physiological conditions and related organic psychiatric disorders (F06–F07); schizophrenia, schizotypal and delusional disorders (F20–F29); disorders of adult personality and behaviour (F60–F69) |
